# Enabling primary healthcare service development with patient participation: a qualitative study of the internal facilitator role in Norway

**DOI:** 10.1017/S1463423623000488

**Published:** 2023-09-27

**Authors:** Ann Britt Sandvin Olsson, Una Stenberg, Mette Haaland-Øverby, Tor Slettebø, Anita Strøm

**Affiliations:** 1 Norwegian National Advisory Unit on Learning and Mastery in Health, Oslo University Hospital, Oslo, Norway; 2 VID Specialized University, Center of Diaconia and Professional Practice, Oslo, Norway; 3 Frambu Resource Center for Rare Disorders, Siggerud, Norway; 4 Oslo Metropolitan University, Faculty of Health Sciences, Oslo, Norway; 5 VID Specialized University, Faculty of Social Studies, Oslo, Norway; 6 VID Specialized University, Faculty of Health Sciences, Oslo, Norway

**Keywords:** Patient participation, facilitation, implementation, health service development, primary healthcare, involvement in research

## Abstract

**Aim:**

To explore how primary healthcare professionals (HCPs) tasked with facilitating primary healthcare service development with patient participation perceived their role.

**Introduction:**

Patient participation in health service development is a recognized means of ensuring that health services fit the public’s needs. However, HCPs are often uncertain about how to involve patient representatives (PRs), and patient participation is poorly implemented. Inspired by the Promoting Action on Research Implementation in Health Services framework, we address the innovation (patient participation), its recipients (PRs, HCPs, supervisors, and senior managers), and its context (primary healthcare at a local and organizational level).

**Methods:**

We conducted semi-structured individual interviews with six HCPs working as internal facilitators in primary healthcare in four Norwegian municipalities. The data were analyzed by applying Braun and Clarke’s reflexive thematic analysis.

**Findings:**

The themes show that to develop primary healthcare services with patient participation, facilitators must establish a network of PRs with relevant skills, promote involvement within their organization, engage HCPs favorable toward patient participation, and demonstrate to supervisors and senior managers its usefulness to win their support. Implementing patient participation must be a shared, collective responsibility of facilitators, supervisors, and senior management. However, supervisors and senior management appear not to fully understand the potential of involvement or how to support the facilitators. The facilitator role requires continuous and systematic work on multiple organizational levels to enable the development of health services with patient participation. It entails maintaining a network of persons with experiential knowledge, engaging HCPs, and having senior management’s understanding and support.

## Introduction

Primary healthcare is under pressure to improve, organize, and deliver high-quality healthcare services (Bergström *et al.*, 2020; Siantz *et al.*, 2021; Walunas *et al.*, 2021). Patient participation is a widely recognized means of ensuring that the public receives good quality healthcare services suited to their needs (Fredriksson and Tritter, 2017; Halabi *et al.*, 2020). Thus, healthcare professionals (HCPs) are called upon to involve individuals, families, and communities in all issues that affect health to establish well-functioning and sustainable primary healthcare services (OECD, 2020; WHO, 2018). However, involving patients and the public in developing or improving health services is a complex intervention (Staniszewska *et al.*, 2012), requiring facilitation (Bergström *et al.*, 2020; Walunas *et al.*, 2021).

In this article, ‘facilitation’ refers to the activities of an internal facilitator: a healthcare professional (hereafter called HCP) within primary healthcare whose responsibility is to lead collaboration between patient representatives (hereafter called PRs) and colleague HCPs of multiple professional backgrounds. The goal is to develop or improve primary health services (see Andreassen, 2017). ‘Patient participation’ is understood as PRs’ active contributions to those processes to ensure that health services answer the public’s needs (Crawford *et al.*, 2002). PRs are persons with experiential knowledge about living with a health challenge. Some PRs represent a patient organization, for example, concerning cancer, dementia, or heart and lung disease. Others are self-selected spokespersons for the patient perspective.

To establish evidence-based practices, experiential knowledge is necessary, in addition to research and professional knowledge (Dawes *et al.*, 2005). However, HCPs report uncertainty about how to facilitate health service development with patient participation (Sandvin Olsson *et al.*, 2020; Boström *et al.*, 2017; Lavoie-Tremblay *et al.*, 2014). For example, HCPs are skeptical of the origin and the meaning content of PRs’ input while simultaneously developing a healthcare service they can approve of professionally (Sandvin Olsson *et al.*, 2022; Renedo *et al.*, 2018). Responding to workplace demands for evidence-based practices, they seek to sort out and incorporate useful and relevant patient experiences to combine them with research and professional knowledge: a complicated task (Renedo *et al.*, 2018). Still, facilitation of health service development is often assigned to HCPs with little or no facilitation experience or skills, who work in under-resourced contexts (Bergström *et al.*, 2020; Walunas *et al.*, 2021).

There are guides on how to facilitate implementing innovations into practice (Damschroder *et al.*, 2009; Harvey and Kitson, 2015; Powell *et al.*, 2015). Also, websites indicate steps to take in the co-production of health services (see, e.g., Involve, 2023), experience-based co-design (see, e.g., The Point of Care Foundation, 2023), or service design approaches to improve health services (see, e.g., Interaction Design Foundation, 2023). Still, there are calls for research on facilitation practices (Hunter *et al.*, 2020; Nguyen *et al.*, 2020; Roberts *et al.*, 2021; Siantz *et al.*, 2021).

Despite being a long-standing international policy (WHO, 1978, 2018), conducting health service development with patient participation remains poorly implemented (Halabi *et al.*, 2020). It seems likely that uncertainties as to how to facilitate such processes may partly be responsible. The gap between global policies and the implementation of health service development with patient participation implies a need to investigate further the facilitator role.

The integrated Promoting Action on Research Implementation in Health Services (IPARIHS) framework provides guidance on navigating the difficulties of implementing research or programs into practice (Hunter *et al.*, 2020; Shoobridge *et al.*, 2021). It identifies facilitation as a key ingredient in implementation processes and indicates that the facilitators’ activities must concern ‘the innovation and the recipients within their local, organizational and wider health system context to enable effective implementation’ (Harvey and Kitson, 2015:14). Highlighting the complexity of implementing innovation, the framework builds on a broad theoretical base, linked to client-centered approaches, theories of innovation and learning, organizational culture, continuous quality improvement, and more (Kitson and Harvey, 2016).

The IPARIHS framework aims to ensure evidence-based practice in healthcare (Harvey and Kitson, 2015). Developing health services with patient participation aims to provide qualitatively good health services suited to the public’s needs (Andreassen, 2017, Tritter, 2009). While these two approaches to enhancing the quality of health services have slightly different focuses, their goals overlap, and facilitation is needed in both types of processes. We thus find that the IPARIHS framework (Kitson and Harvey, 2016) may be applied retrospectively to explore the facilitator role in health service development that incorporates experiential knowledge through patient participation. We investigate facilitation by addressing the IPARIHS framework’s (Harvey and Kitson, 2015) concepts of innovation, recipients, and context. In our study, *innovation* corresponds to patient participation in developing health services. The *recipients* are understood as those affected by the implementation of patient participation in the development of health services: supervisors and senior managers on the organizational level, and HCPs and PRs involved in health service development. The implementation *context* is primary healthcare in Norway.

### The Norwegian context for primary healthcare service development

Norwegian welfare is organized mainly as a public service. It is the municipalities’ responsibility to ensure democracy and provide efficient primary healthcare services of high quality (Andersen, 2020). Policies of including patient participation in the development of primary healthcare services are emphasized in white papers (Norwegian Ministry of Justice and Public Security, 2012); Norwegian Ministry of Health and Care Services, 2015) and laws. *The Health and Care Services Act § 3-10* (in Norwegian: Helse- og omsorgstjenesteloven, 2011) grants PRs the right to be heard in health service development and confirms HCPs’ duty to facilitate the participation of PRs. Also, the *Regulations on Management and Quality Improvement in Health and Care Services* (in Norwegian: Forskrift om ledelse og kvalitetsforbedring i helse- og omsorgstjenestene, 2016) underlines the municipalities’ duty to evaluate and apply experiential knowledge when improving the primary healthcare services.

The Norwegian government has supported the establishment of municipal centers that provide low-threshold community interventions supporting efforts to live a healthier lifestyle or better cope with health challenges (Fønhus and Dalsbø, 2022). The centers are called Healthy Life and Coping Centers (in Norwegian: Frisklivs- og mestringssentre), and around 30 of them offer such activities, either on their own or in collaboration with neighboring municipalities. The centers commonly have one or two HCPs in full- or part-time positions. They are responsible for leading processes to develop, implement, and evaluate interventions, self-management programs, and health services in primary healthcare (Norwegian National Advisory Unit on Learning and Mastery in Health, 2020). In this way, the HCPs function as internal facilitators of primary healthcare development with patient participation.

## Aim

To advance existing knowledge about patient participation, this article explores how HCPs tasked with facilitating primary healthcare service development with patient participation in Norway perceived the facilitator role.

## Methods

This is an exploratory, qualitative study. It is founded on an interpretivist/constructivist paradigm that aims to understand human experience and regards reality as socially created (Mackenzie & Knipe, 2006). In line with the American Psychological Association’s guidelines concerning bias-free language (APA, 2022), we have used the gender-neutral pronoun ‘they’ in the singular to refer to the participants.

### Involvement in research

This study is part of a broader PhD project with a bottom-up approach to developing knowledge. We first conducted a scoping review (Sandvin Olsson *et al.*, 2020), addressing the potential impact of adult patient participation and identifying the need for new research. Then we applied multiple qualitative methods to explore the perspectives on primary healthcare service development with patient participation from those involved. The current article explores the facilitator perspective based on individual interviews. One article (Sandvin Olsson *et al.*, 2022) applies focus groups to address the HCPs’ perspective, and another article (Sandvin Olsson *et al.*, 2023: Manuscript under review) uses focus groups and individual interviews to explore the perspective of involved PRs. Most of the authors have extensive experience of health service development with patient participation, and long experience with involvement in research.

This study relies on involvement in research based on a substantive value system to enhance research quality, relevance, and credibility (Gradinger *et al.*, 2015). In line with Malterud and Elvbakken (2020), a co-researcher is included as a full research team member. The co-researcher has experience as a patient in primary and specialized healthcare and patient organization activities. She is educated in how to take part in designing and carrying out research while asserting a patient perspective in the process. When needed, she had the support of the research team members.

The study further involved a mixed advisory panel (herafter called `the panel´) comprised of three PRs and three HCPs. The PRs have extensive experience collaborating with HCPs to develop self-management courses and patient education and improve health services. The HCPs have extensive experience in facilitating the development of self-management courses, patient education, and improvement projects with patient participation. When needed, the panel members had the support of the first author and the co-researcher.

Conducting involvement in research implies integrating multiple perspectives in the research process (Rycroft-Malone *et al.*, 2016). It further implies an understanding that knowledge is socially generated, that the involved contribute to shaping the research process and its findings, and that the presuppositions of those involved matter. Representing different backgrounds and types of knowledge is considered a strength. Upon establishing the research team and the panel, the members engaged in debates to establish their roles in the PhD project’s knowledge production.

### Setting

Norwegian municipal Healthy Life and Coping Centers constitute our setting to explore the facilitators’ perception of their role in health service development with patient participation. The facilitators’ way of working is loosely outlined in the manual ‘The Standard Working Method for the Learning and Coping Centers – the 2011 Version’ (Norwegian National Advisory Unit on Learning and Mastery in Health, 2011). The systematic incorporation of dialog and patient participation in the four phases of the Plan-Do-Study-Act (PDSA) Cycle (see Langley *et al.*, 2009) are key aspects (Hvinden, 2012). To illustrate, the facilitators typically invite HCPs with relevant backgrounds and PRs from relevant patient organizations and facilitate the planning (Plan) of specific health services or programs. Ideally, the HCPs and PRs are also involved in its implementation (Do). This allows them to provide their input during the evaluation phase (Study) and make the necessary improvements (Act) before the health service or program is put into practice.

Depending on its aim, some processes may require the facilitators to lead the collaboration between HCPs and PRs over the course of six months and multiple meetings before they have produced a result suited to the public’s needs. Other processes may only entail a meeting or two over a couple of hours with a few designated HCPs and PRs. Examples of such processes can include: the development and implementation of self-management programs concerning how to cope with living well with chronic health challenges; the development of patient education concerning non-communicable diseases for informal carers; the improvement of primary healthcare wards’ communication with patients, users, and informal carers; and adapting and implementing a government program aimed at improving the public’s mental health into primary healthcare.

### Sampling strategy

Our colleagues at the Norwegian National Advisory Unit for Learning and Mastery in Health helped make a list of centers with at least one HCP whose role entailed being a facilitator. Based on that list, we invited municipalities that vary in population size, their application of patient participation in primary healthcare service development, and location. All agreed to participate, and each appointed a contact person.

The contacts recruited the participants. They were purposively sampled to ensure they had the required experience (Etikan *et al.*, 2016; Malterud *et al.*, 2016). We also ensured they had different degrees of experience, aiming for rich descriptions. Two municipalities’ centers employed two HCPs with different degrees of facilitator experience. We chose to interview both of these facilitators. All those invited agreed to participate. A total number of six facilitators from four municipalities’ Healthy Life and Coping Centers participated in this study.

### Data generation

The first author and the co-researcher participated in a health service development meeting in each of the municipalities. We did so to become familiar with the place, its culture, and people, and to explore what was significant to investigate (Fangen, 2010; Hammersley, 2006). Furthermore, to establish a good relationship with the contacts and ensure the purposive sampling of participants (Malterud *et al.*, 2016).

We needed to understand each facilitator well to answer our research questions. As individual interviews allow the participants to elaborate on and clarify their situated meaning (Brinkman and Kvale, 2015), we chose to conduct individual semi-structured 90-minute interviews.

The interview guide was drafted by the first author. The interview guide questions were developed based on the data available from the PhD project at the time. These included (1) the co-researcher and first author’s participation in health service development meetings in each of the four municipalities, (2) transcripts from four focus groups with HCPs (see Sandvin Olsson *et al.*, 2022), (3) transcripts from four focus groups with PRs (see Sandvin Olsson *et al.*, 2023: Manuscript under review), and (4) our scoping review (see Sandvin Olsson *et al.*, 2020).

The interview guide draft was finalized after discussions with the panel. The co-researcher and the panel contributed to enhancing the guide’s language. They added the following questions: ‘In what situations is professional knowledge more valuable than experiential knowledge, or vice versa?’ and ‘Please reflect on whether you are more likely to act on suggestions from a PR you know and trust rather than one you are unfamiliar with’. While the guide remained the same throughout the interviews, some issues were investigated more thoroughly in some interviews than others. This was done to ensure that the generated data would answer the research questions adequately (Malterud *et al.*, 2016; O’reilly and Parker, 2013).

The first and second authors conducted a pilot interview before the first author conducted the individual interviews with the six participants. Each participant was encouraged to speak freely and share their experience in response to the interview guide’s topics. Topics included the participants’ professional background, the context of primary healthcare service development, the meaning and practice of patient participation, perceived functions as a facilitator, expectations of those involved, and the potential impact of patient participation. After each interview, the first author noted interview reflections in the reflexive journal before discussing these and the generated data with the co-researcher and the last author. After conducting six interviews and familiarizing ourselves with the data, we shared the anonymized transcripts with the other authors. Reviewing and debating the transcribed interviews’ content in relation to our research interests and from our various perspectives, we found that we could answer the research question adequately.

### Analysis

The interviews were transcribed verbatim in Norwegian, and pauses were identified. The quotations selected for this article were clarified without affecting their meaning, translated, and back-translated by a professional language consultant (Braun and Clarke, 2013). We used NVivo to sort the data (QSR International, 2020).

A thematic analysis enables the researcher to systematize and arrange patterns of meaning or experience across data sets (Braun and Clarke, 2013). Therefore, the data were analyzed following the six-phase process for reflexive thematic analysis: familiarization with the data, coding, generating themes, reviewing themes, defining and naming themes, and writing up the study (Braun and Clarke, 2019).

To become familiar with the data, the first and last authors and the co-researcher read and recorded ‘noticings’: our impressions, ideas, and associations in response to the transcribed data. We highlighted text excerpts and recorded corresponding ‘noticings’ in our reflexive journals. We used them to discuss what perspectives and presuppositions we brought into our interpretation of the data (Braun and Clarke, 2013). Through the process, we better understood our preconceived notions and that different backgrounds provide different interpretations of the data.

The discussion provided a background against which the first author openly coded each interview’s data transcript, adding, developing, and renaming codes in English. When the relevant data were coded, the first author generated a Codebook applying NVivo (QSR International, 2020). It included all codes and their corresponding quotations from the data set. Reviewing the codebook, the first and last authors and the co-researcher scrutinized the codes until we were clear about their names and meaning. Thereafter, we looked for patterns of shared meaning across the interviews (Braun and Clarke, 2019). After the discussion, the first author renamed, re-coded, and added new codes in NVivo. Against this background, the first author developed the initial themes, constantly going back and forth between the six phases.

To facilitate the panel’s involvement in the initial analysis, the first author translated themes and codes into Norwegian, added the corresponding quotations, prior to sending them the document. They were asked to record their immediate understanding of each theme’s name and, next, how the quotations reflected the themes’ names. In the meeting, we scrutinized the themes by reviewing and debating their differing reflections. The process contributed to renaming themes and selecting a sample of the most appropriate quotations to illustrate each theme’s content.

The first author edited the theme names according to the co-researcher and panel’s input and translated them into English. A document constituting the English theme names and codes and the corresponding samples of Norwegian quotations was sent to the authors. A week later, all the authors reviewed and discussed the themes’ names, codes, and quotations. The process yielded adjustments to the themes’ names and started clustering themes. The further clustering of themes evolved through the first author’s repetition of the six sequential phases (Braun and Clarke, 2019).

This approach contributed to the incorporation of reflections from multiple perspectives and a rich analysis. The analysis continued until the final manuscript had been drafted. The developed themes and subthemes are displayed in Table 1.

**Table 1.** Developed themes and subthemes describing the facilitators’ perceived role in enabling primary healthcare service development with patient participation

Themes	Establishing a network of patient representatives	Encouraging healthcare professionals to engage with patient participation	Calling upon senior managers and supervisors to take responsibility
Subthemes	Locating, selecting, and training suitable patient representatives	Promoting patient participation	Tackling barriers to patient participation
	Establishing good relations to ensure patient representatives’ future participation	Locating and engaging healthcare professionals	Countering senior managers’ and supervisors’ hesitancy toward patient participation

### Techniques to enhance trustworthiness

The trustworthiness of research relies on a study’s credibility, transferability, dependability, confirmability, and the researchers’ reflexivity (Lincoln and Guba, 1985). To strengthen credibility, the first author and the co-researcher both familiarized themselves with the setting and identified important topics to explore. As reflexivity helps safeguard against research bias, we kept reflexive journals and debated our preconceptions, researcher roles, interpretations, and the findings. Investigator triangulation was ensured through the discussion of codes and by involving the mixed advisory panel and all authors in the analysis. To enhance transferability, we have carefully described the study’s setting, sampling strategy, participants’ characteristics, and data generation. For transparency, we followed appropriate guidelines for assessing the quality of thematic analysis research (Braun and Clarke, 2021) and the stages in reflexive thematic analysis (Braun and Clarke, 2019). This article was informed by ‘Standards for Reporting Qualitative Research: A Synthesis of Recommendations’ (O’Brien *et al.*, 2014).

## Findings

Based on the analysis, three themes were developed concerning the facilitators’ perceived role in enabling primary healthcare service development with patient participation (see Table 1).

The first theme indicates facilitators must establish and sustain a network of suitable PRs. The second theme suggests they must promote patient participation within their organization and engage HCPs favorable to patient participation. The third theme implies they must convince their superiors that patient participation is useful to get support and receive the necessary resources.

In the following, we first present the participants’ characteristics. Then we present the themes and their respective subthemes.

### Participant characteristics

The contacts recruited six HCPs identified as cisgender women who had acted as facilitators.

The participants’ experiences facilitating health service development with patient participation differed. One had over ten years’ experience, two had between six and eight years, and three had two years. However, they had all initiated and led primary healthcare service development more than four times. Their professional backgrounds were in nursing, occupational therapy, psychology, and physical therapy. They had different professional roles and responsibilities: one healthcare division manager, two service level leaders, and three service level coordinators. Their age range was 39–62 years, with a mean age of 50 years.

### Establishing a network of PRs

This first theme concerned the facilitators’ efforts to establish and sustain a network of suitable PRs who were willing to be involved when needed. To do so, they must locate, select, and train PRs before involving them in primary healthcare service development. Furthermore, the facilitators must ensure good relations between the PRs and the municipality’s senior management to maintain the PRs in their network.

#### Locating, selecting, and training suitable PRs

The facilitators acknowledged that PRs were a scarce resource. They also acknowledged that working alongside PRs with diverse attitudes, expectations, and collaboration skills could pose difficulties. Still, they were dependent on PRs’ participation to conduct health service development. Therefore, they went to great lengths to locate suitable PRs to include in their network.

It is all about how they behave. The language they use, and their attitudes toward those they will be meeting with. It’s also about humility, which, of course, goes for all involved. (Facilitator A)

The facilitators tried to find PRs by participating in conferences or in seminars at the local hospital, actively recruiting participants from self-management programs, or accepting previous participants who volunteered. They also sought out individuals with prominent roles in patient groups. Alternatively, they asked patient organizations to provide PRs, who were willing to be involved. However, some facilitators had negative experiences of this strategy.

We need to know them first. (.) We have experience with contacting patient organizations. (.) Then we’re at the mercy of the person they send, and we don’t know if this person is someone who can speak on behalf of many, or is this person someone who can’t see beyond their own suffering. (Facilitator B)

The quotation exemplifies an important issue stressed by the facilitators. They needed PRs who understood whom they represented and their role to ensure constructive collaboration. It would ease their job when facilitating collaboration between HCPs and PRs.

To avoid complications, some facilitators met with all potential PRs before involving them. They asked the PRs about their motives for participating, their experience, and what was important to them. Despite their efforts to involve suitable PRs, the facilitators sometimes found that PRs raised private issues or acted disrespectfully toward other members involved with the service development. Then it was the facilitators’ task to take the PRs aside and gently suggest they behave differently to contribute to constructive collaboration.

One facilitator pointed out that some patient organizations demand that their PRs undergo training for their role before participating. They said it made their selection of PRs easier, as they could also pose those demands. The facilitators agreed that it was their task to clarify the PRs’ role. However, they emphasized that participating in health service development also meant that the PRs had responsibilities to fulfill.

They have said yes to taking responsibility. Because it is a responsibility, it’s a job that needs to be done. Participating isn’t just for fun. This needs to be clear. (Facilitator A)

The facilitators looked for PRs with specific attributes and skills. In particular, the ability to see issues from a meta-patient’s perspective. However, they understood that it could sometimes be difficult for the PRs to keep that perspective.

It’s important that they have their own experience, but at the same time, that they know they represent the experience of many. However, they can slip in and out of that perspective, depending on the issue addressed, or their current health situation. That’s just the way it is. (Facilitator D)

The facilitators also said that they tried to involve PRs with diverse experience, some knowledge about the municipal system, and with enough self-assurance to offer ideas and discuss issues in an unassuming and respectful manner. However, due to PRs’ health challenges, they could be indisposed from participating for periods. Thus, the facilitators tried to maintain a few PRs in their network. It also enabled them to pick the PR with the most relevant background to participate in health service development.

#### Establishing good relations to ensure PRs’ future participation

The facilitators found that senior managers and supervisors often failed to recognize the importance of making PRs feel appreciated and contribute to PRs choosing to be involved. The facilitators found their leaders’ lack of concern for the PRs’ future participation frustrating, as they worked hard to include PRs and maintain their network.

One facilitator described feeling deeply embarrassed on several occasions because their supervisor had not responded to patient organizations’ repeated inquiries. This negatively affected the relationship and collaboration with the patient organizations. Another described spending time mediating between senior managers and PRs to ensure good relationships and the future participation of PRs.

I go down to my municipal director and ask why he hasn’t responded to the email from the patient organization. (.) He says he’s sorry and explains that it has been so busy with the reorganization. Then I report this back to the PR, making sure that the PR understands that the lack of response has nothing to do with them. (Facilitator F)

To foster good relations with their network of PRs, the facilitators would have liked to keep them engaged when they were not actively involved with health service development. However, the facilitators had little time to organize this.

### Encouraging HCPs to engage with patient participation

The second theme addressed the facilitators’ role in familiarizing their colleagues with patient participation and encouraging them to collaborate with PRs. Their efforts entailed promoting patient participation in the organization and locating and engaging HCPs to be involved with health service development.

#### Promoting patient participation

When describing their role, the facilitators reflected on how they had come to see the importance of patient participation; they had developed their understanding by practicing it.

I think it’s all about having experience and knowledge. Because it wasn’t that I didn’t want to involve PRs, but I just didn’t understand it like I do now. (Facilitator D)

The facilitators said that many HCPs believed they were practicing patient participation. However, in their view, HCPs often did so only partially. With their own experience in mind, the facilitators applied several means to raise engagement about patient participation in their organizations. For example, they said they used humor and irony to ease any concerns or worries HCPs may have about including PRs in developing health services.

The facilitators also teamed up with PRs to promote patient participation. In meetings with HCPs, they conveyed what the collaboration meant to them, emphasizing its positive impact. One facilitator emphasized that arranging for HCPs and PRs to meet was the most important activity in terms of promoting patient participation, and that consistently speaking positively about the patient organizations’ competence was essential. They found that this helped their colleagues think of the patient organizations as resources and potential collaborators.

#### Locating and engaging *HCP*s

The facilitator role entailed locating and engaging HCPs who could contribute to constructive health service development with good results. The facilitators had fewer criteria for HCP involvement than they had for the PRs. However, they were clear that the HCPs must be respectful, recognizing that collaborating with PRs was sometimes difficult for the HCPs.

You have to be sure of yourself to be open and handle feedback well. Perhaps it depends on how much experience you have – and your attitude. Or knowing that you don’t have all the answers, and can learn something from the PRs. (Facilitator C)

The facilitators tried to involve HCPs who appeared confident in their professional roles and dared to question and discuss the PRs’ input. However, when the facilitators found suitable HCPs, the HCPs’ supervisors would not always let them take time away from their regular duties. Thus, the facilitators sometimes had to persuade the HCPs to participate in health service development on top of their other responsibilities.

You have to find what motivates them to get involved and keep the motivation alive. (Facilitator E)

Still, the facilitators felt that it was their superiors’ job to establish routines that enabled HCPs to participate as part of their regular workday.

### Calling upon senior managers and supervisors to take responsibility

The third and last theme was the facilitator’s role in encouraging senior managers and supervisors to take responsibility for systematic and long-term implementation of patient participation in primary healthcare. The facilitators tackled several barriers to patient participation, including how to fund the PRs’ participation. Furthermore, they tried to counter their leaders’ hesitancy to involve PRs.

#### Tackling barriers to patient participation

All facilitators but one experienced that their senior managers were not focused on implementing patient participation.

No leader says that patient participation is not important. However, how they support it, focus on it, and create structures for it, varies. (Facilitator D)

Only one facilitator had received a budget for participatory activities from their senior manager. The facilitators saw payment as a token of respect and an acknowledgment of the PRs’ work. Still, paying the PRs was a true challenge.

We don’t have a budget for patient participation. Still, hospitals and local and national guidelines demand it. However – they fail to mention that this costs money. (Facilitator B)

Some facilitators felt responsible for keeping costs down and refrained from hosting meetings where PRs should naturally have been included. They also involved fewer PRs than they preferred. This meant that they missed out on important input, but the facilitators felt they had no choice. One facilitator described spending a lot of time applying for funds. Another gave the PRs jobs within the health and social services; they found this an excellent strategy for ensuring the user voice was present in several primary healthcare settings.

#### Countering senior managers and supervisors’ hesitancy toward patient participation

The facilitators were challenged by their superiors’ attitudes about patient participation. They asserted that their superiors should be role models and actively implement patient participation in primary healthcare. However, only one of the facilitators found that their superiors shouldered this responsibility. One facilitator stressed the need for them to be genuinely curious about the PRs’ input and value the user voice.

We need to see patient participation as an opportunity and be genuinely interested in finding that which is important and hidden amidst the chatter of the interest organizations and the personal stories that suddenly get told. HPCs, leaders, and politicians also say a lot of bullshit. Still, we so easily dismiss the user voice as not being valuable enough. (Facilitator E)

Another facilitator experienced that their superiors’ tokenistic attitudes reflected on her; they felt ignored when suggesting involving PRs in meetings. They said it often made them refrain from asserting that PRs should be present. However, they found ways to work around their superiors’ hesitations to involve PRs.

Sometimes we bring the PRs along to meetings, as one of the employees, without asking if it’s OK. Nobody sees any difference if there is any. (Facilitator F)

The facilitators said that PRs noticed the senior managers and supervisors’ attitudes concerning patient participation. The PRs were careful to invest their time and energy where they could make a difference. Some facilitators feared their superiors’ lack of responses to patient organizations’ requests would make PRs unwilling to get involved.

To improve their supervisor’s attitude about patient participation and gain support, one facilitator had systematically brought the supervisor’s questions about service quality to their trusted group of PRs to get their opinion.

After a while, I noticed a difference in my leader. When I again and again took ideas to the PRs and came back with relevant responses, without telling my opinion, my leader became curious and began to see the value in patient participation. My leader stopped ignoring them. (Facilitator E)

The facilitator was convinced that the supervisor would not have shown the same willingness toward patient participation if they had not demonstrated its benefits. However, it required much time and effort. Correspondingly, another facilitator feared starting from scratch if a new supervisor was hired.

Not all leaders really understand what patient participation is. They need to practice it a bit before they understand it. (Facilitator D)

This facilitator indicated that if the new supervisor was not familiar with patient participation, they would quit their job. They emphasized that leaders in primary healthcare must know what patient participation is and entails, have experience practicing it, and understand that it takes time to implement. Another facilitator reflected that after 10 years of work to implement patient participation in primary healthcare, the practice was still fragile and required constant attention to maintain the support of PRs, HCPs, supervisors, and senior management.

## Discussion

This article elucidates facilitators’ perceptions of their role in facilitating primary healthcare service development with patient participation. The three identified themes suggest that the facilitator role included working continuously and systematically on multiple organizational levels to implement patient participation in developing or improving health services. Firstly, facilitators must establish and maintain a network of PRs with relevant skills. Secondly, they must promote patient participation within their organization and engage suitable HCPs favorable to patient participation. Thirdly, they must convince their superiors that patient participation is useful to get support and necessary resources.

As mentioned in the Introduction, the IPARIHS framework (see Kitson and Harvey, 2016) (hereafter called the framework) emphasizes facilitation as a key ingredient in successful implementation of a desired change or outcome, which the framework calls the innovation (Hunter *et al.*, 2020; Shoobridge *et al.*, 2021). The framework can contribute to exploring the facilitator role in health service development with patient participation. Thus, we have used the framework to organize and inform our discussion. First, we address facilitation in light of the *innovation* (patient participation in health service development), then its *recipients* (PRs, HCPs, supervisors, and senior managers), and last, its *context* (primary healthcare on a local and organizational level).

### The innovation

For patient participation to be successfully integrated into practice, it needs to be well understood by those involved in the implementation process (Harvey and Kitson, 2015). However, our findings suggest that patient participation has some characteristics that may make it hard to grasp and, thus, implement.

Studies have shown that patient participation can be understood differently within and between organizations, as well as between individuals within the same organization; this can cause confusion (Bombard *et al.*, 2018; Halabi *et al.*, 2020). This may partly explain why the HCPs knew they should involve PRs in health service development (Rycroft-Malone *et al.*, 2015), but were uncertain about what it was (Forbat *et al.*, 2009; Martin and Finn, 2011), and how to integrate PRs’ input into health service development (Renedo *et al.*, 2018; Sandvin Olsson *et al.*, 2022). Our findings suggest that the facilitators needed to collaborate with the PRs to learn what patient participation entailed and could accomplish. Moreover, while their superiors and HCPs believed they were practicing patient participation, the facilitators found they were not – yet another indicator that they did not fully understand what patient participation entailed.

Halabi and colleagues suggest training HCPs to enhance the application of patient participation (Halabi *et al.*, 2020). Those facilitating collaboration and co-production of knowledge in healthcare need knowledge about patient participation, how to lead collaborative processes, as well as facilitation methods and techniques (Rycroft-Malone *et al.*, 2015). We believe there are similar needs in health service development and improvement. Our findings suggest that the implementation of patient participation in health service development would benefit from first-hand experience of constructive collaboration with PRs. This can be achieved in existing undergraduate and postgraduate education for HCPs and in professional development programs for supervisors and senior managers.

### The recipients

From a facilitator’s perspective, recipients are those who ‘will be directly involved in and affected by the implementation process’ (Harvey and Kitson, 2015:53). Implementation of patient participation in the development of health services affects supervisors and senior managers, HCPs, and the recruited PRs.

Recipients’ characteristics and attitudes can hinder or facilitate the implementation process (Kitson and Harvey, 2016). Studies have identified that HCPs have concerns about the legitimacy of PRs’ knowledge and power imbalances between them (Ayton *et al.*, 2017; O’Shea *et al.*, 2019), about receiving unpredictable input from PRs (Ocloo *et al.*, 2021), as well as having too little time and resources to be involved in addition to their regular duties (Ayton *et al.*, 2017; Lavoie-Tremblay *et al.*, 2014). It makes sense that the facilitators tried to find ‘good’ HCPs and PRs whom they trusted would contribute to a constructive process with valuable outcomes. The facilitators’ selection of ‘good PRs’ can imply a skepticism toward the legitimacy of PRs’ knowledge. However, it can also be understood to highlight the complexity faced by the facilitators when trying to implement patient participation.

Implementing an innovation often takes convincing colleagues of its usefulness (Harvey and Kitson, 2015). The IPARIHS framework thus encourages facilitators to identify clinical champions to assist with the implementation (Harvey and Kitson, 2015). Involving ‘good PRs’ can be seen as the facilitators’ attempt to get help in building a rapport for patient participation and demonstrating its usefulness to their colleagues.

Renedo and colleagues found that HCPs welcome only patient experience that they perceived as useful (Renedo *et al.*, 2018); this is in line with our findings. For instance, the facilitators asked the PRs to provide only a bird’s eye view of their illness experience. On the other hand, studies have shown that PRs tailor their experience to be acceptable to HCPs and to influence the processes (Martin *et al.*, 2018; Renedo and Marston, 2011). The facilitators’ preference for ‘good PRs’ can thus raise questions regarding whose voices, experience, and knowledge count and whose do not.

O’Shea and colleagues found that HCPs‘ practice of patient participation had layers that enabled or hindered PRs’ capacity to influence (O’Shea *et al.*, 2019). The HCPs decided whom to place in what position, decisions largely driven by the value that the HPCs placed on the PRs’ knowledge (O’Shea *et al.*, 2019). Therefore, some PRs had higher status than others, though never equal to the HCPs’, as professional knowledge consistently ranked highest (O’Shea *et al.*, 2019). Consistent with these results, the facilitators in this study trusted some PRs more than others.

Martin and colleagues argue that PR recruitment can actively exclude PRs whom the facilitators see as not having the correct input to share (Martin *et al.*, 2018). They emphasize that well-meaning patient participation efforts can sometimes thwart the original intentions of patient participation (Martin *et al.*, 2018). Facilitators must be aware of such downsides and avoid them (Martin *et al.*, 2018). Though we agree, our findings can provide nuance about why facilitators select some PRs and not others by identifying challenges that facilitators face when organizing patient participation in an organization unprepared for it.

Our findings showed that one facilitator felt devalued by their superiors when suggesting involving PRs in meetings. Drawing on O’Shea and colleagues’ findings, indicating that HPCs valued professional over experiential knowledge (O’Shea *et al.*, 2019), it appears that the facilitator’s workplace status was reduced by their acknowledgment of experiential knowledge. Perhaps facilitators should be made aware of issues that may negatively affect their position among colleagues, and how to tackle such incidents if they arise.

### The context

When facilitating the uptake of an innovation, the organizational culture and leadership play an important role (Rycroft-Malone *et al.*, 2018). A supportive managerial relationship is crucial for the facilitator to implement change successfully (Roberts *et al.*, 2021; Seers *et al.*, 2018); this includes showing direction and vision supporting the implementation process (van der Zijpp *et al.*, 2016). Managerial support from the very start is vital for acquiring everything required for productive facilitation, and a lack of support will hinder progress (Rycroft-Malone *et al.*, 2018; van der Zijpp *et al.*, 2016). These findings resonate with ours. Only municipalities with a history of patient participation in health service development participated in our study. Nevertheless, except in one municipality, the facilitators found their senior managers unfamiliar with patient participation. This hindered its implementation. Furthermore, only one facilitator had a budget for patient participation. The others reported lacking funds to pay PRs and protected time for HCPs to be involved. This ultimately limited patient participation.

Studies imply that the relationship between facilitators and managers is neglected and needs more focus to ensure successful implementation processes (van der Zijpp *et al.*, 2016). Our findings further indicate that the relationship between managerial leadership and PRs in the facilitators’ network is also neglected. The facilitators spent time and energy mediating. When senior managers or supervisors did not, for instance, respond to inquiries from PRs, it affected the facilitators’ collaboration with the PRs.

Studies show that it is not uncommon to have managers’ support for a project ‘in principle’ only to find out that it was not backed up with resources and tangible support (Hespe *et al.*, 2022, Murray *et al.*, 2022). The lack of understanding and support for patient participation from their supervisors was described as the most challenging aspect of the facilitator role. It led some to consider quitting their job. As patient participation remains poorly implemented (Halabi *et al.*, 2020), perhaps senior management and supervisors should consider participating in educational programs to better understand what patient participation is and its potential impact. The training could include participating in service development with PRs and discussing what values patient participation could contribute to their organization.

Regarding leadership roles in implementing research into practice, Harvey and colleagues found ‘a need to maintain a balance between the mechanisms of managing and monitoring performance versus facilitating critical questioning and reflection in and on practice’ (Harvey *et al.*, 2019:29). Accordingly, we find that successfully implementing patient participation when developing or improving primary healthcare services requires collective responsibility, with continuous efforts from facilitators, supervisors, and senior managers.

Limitations related to social desirability and reporting bias in participants’ self-presentation in the individual interviews should be considered (see Bergen and Labonté, 2020). The author conducting the interviews represented the Norwegian National Advisory Unit on Learning and Mastery in Health at Oslo University Hospital. Thus, participants may have hesitated to reveal negative experiences regarding health service development with patient participation. However, participants indeed shared difficulties, negative, and positive experiences. A strength of this study is the credibility of its interpretations, derived from multiple perspectives: a mixed advisory panel of HCPs and PRs, and diverse authors (including a co-researcher with experiential knowledge). The use of reflexive thematic analysis, enabling closeness to the empirical data during a comprehensive iterative analytical process (Braun and Clarke, 2021), represents another strength.

## Conclusion

This article supplements the literature with a detailed account of the context surrounding the facilitation of primary healthcare service development with patient participation. By applying the IPARIHS framework (see Kitson and Harvey, 2016), it contributes to the discourse concerning what is required to implement patient participation when developing or improving health services. Our findings demonstrate that the facilitator role requires continuous and systematic work on multiple organizational levels, especially since senior management and supervisors appear not to fully understand the potential of patient participation or how to support facilitators’ work. Implementing patient participation as part of health service development or improvement must be a shared responsibility of facilitators, supervisors, and senior management. This article confirms that to enable patient participation when developing or improving health services, facilitators must maintain a panel of persons with experiential knowledge, engage HCPs, and have senior management’s understanding and support.

Future research could explore senior managers’ perceptions of resources required for an organizational commitment to patient participation when developing health services. Future research could also investigate the implications of contextual factors to senior management’s support for patient participation in health service development.

This study’s findings have implications for practice, education, and research related to application and implementation of patient participation in health service development. It expands knowledge about what it entails to conduct health service development with patient participation. It broadens our understanding of the skills, expertise, and support facilitators need to implement patient participation in primary healthcare and conduct primary healthcare service development with patient participation. The study additionally implies how the knowledge base of patient participation could be advanced.

## Data Availability

The data are not publicly available due to privacy restrictions. The data that support the findings are available from the corresponding author upon reasonable request.
